# Detecting Fifth Metatarsal Fractures on Radiographs Through the Lens of Smartphones: The FIXUS AI Algorithm

**DOI:** 10.7759/cureus.91284

**Published:** 2025-08-30

**Authors:** Atta Taseh, Aaditya Shah, Mani Eftekhari, Alexandra Flaherty, Alireza Ebrahimi, Sumner Jones, Varun Nukala, Ara Nazarian, Gregory Waryasz, Soheil Ashkani-Esfahani

**Affiliations:** 1 Foot and Ankle Research and Innovation Laboratory (FARIL), Mass General Brigham, Harvard Medical School, Boston, USA; 2 Musculoskeletal Translational Innovation Initiative, Carl J. Shapiro Department of Orthopaedic Surgery, Beth Israel Deaconess Medical Center, Harvard Medical School, Boston, USA; 3 Foot and Ankle Division, Department of Orthopaedic Surgery, Mass General Brigham, Harvard Medical School, Boston, USA

**Keywords:** ai and machine learning, artificial intelligence (ai), clinical decision support, foot and ankle fracture, orthopedic surgery research, smartphones as a medical device

## Abstract

Introduction

Fifth metatarsal (5MT) fractures are common but challenging to diagnose, particularly with limited expertise or subtle fractures. Deep learning shows promise, but faces limitations due to image quality requirements. This study develops a deep learning model to detect 5MT fractures from smartphone-captured radiograph images, enhancing the accessibility of diagnostic tools.

Methods

A retrospective study included patients aged ≥18 with 5MT fractures (n = 1,240) and controls (n = 1,224). Radiographs (anteroposterior, oblique, and lateral) from Electronic Health Records (EHRs) were obtained and photographed using a smartphone (SP), creating a new dataset. Models using ResNet-152V2 were trained on EHR, SP, and combined datasets, then evaluated on a separate SP test dataset.

Results

On validation, the SP model achieved optimal performance (area under the receiver operating characteristic curve, or AUROC: 0.99). On the SP test dataset, the EHR model’s performance decreased (AUROC: 0.83), whereas the SP and combined models maintained high performance (AUROC: 0.99).

Conclusion

SP-specific deep learning models effectively detect 5MT fractures, suggesting their practical utility in resource-limited (RL) settings.

## Introduction

Metatarsal fractures are among the most common foot fractures, with an incidence rate of 75.4 per 100,000 people per year. Fifth metatarsal (5MT) fractures account for more than 80% of single metatarsal fractures [1]. These fractures can be categorized into three zones: Zone 1 occurs at the proximal tubercle and is typically treated non-operatively, whereas Zone 2 (Jones fractures) and Zone 3 (proximal diaphyseal fractures) require more detailed assessment for appropriate clinical decision-making [2]. The specific anatomy of the arteries supplying the 5MT bone makes it prone to bone union complications, necessitating meticulous diagnosis and treatment [3]. Diagnosis is based on physical examination and plain radiographs - anteroposterior, lateral, and oblique views - which confirm the fracture location and type [4-8]. 

The delay in the treatment process gives rise to complications like malunion and increases patients’ chances of long-term morbidity [9,10]. This is a frequent scenario for malpractice claims against clinicians, which can happen in high-pressure settings such as emergency departments, as well as in resource-limited (RL) settings [11,12]. While seeking second opinions through teleradiology has been effective in reducing error rates, the infrastructural demands of implementing a Picture Archiving and Communication System (PACS) present significant challenges in RL settings [13]. The literature highlights key issues, including unstable power and network connections, limited image storage capacity, and insufficient knowledge of information technology in these medical settings [14]. Smartphone (SP) communication has emerged as a practical alternative for obtaining second opinions, proving to be promising despite the reduced quality of radiological images [15]. However, the shortage of radiologists in RL places remains a critical issue [13]. Given the advances in artificial intelligence (AI) and fracture detection, SP cameras could become a valuable tool in aiding clinical decision-making, improving accessibility to expert input, and facilitating faster data transfer and communication [16-18].

In this study, we hypothesize that a deep learning AI algorithm trained on SP images of radiographs will achieve superior performance in detecting 5MT fractures, compared to conventionally trained models on high-quality images. Validating this hypothesis through rigorous testing would establish a foundation for potentially leveraging AI technology to enhance diagnostic capabilities in RL or high-pressure settings.

This article was previously posted to the medRxiv preprint server on July 18, 2025.

## Materials and methods

Study design and population

After obtaining approval from the Institutional Review Board (IRB) (approval no. 2023P003483), a retrospective case-control study was conducted at Massachusetts General Hospital (Boston, USA), including data from three hospitals. Due to the retrospective nature of the study, informed consent was waived by the IRB. The study was conducted as part of the FIXUS-AI project, a program in the Department of Orthopaedic Surgery at Massachusetts General Hospital dedicated to designing, validating, and deploying AI solutions to improve orthopaedic patient care and clinical workflows (https://fixus.mgh.harvard.edu).

The International Classification of Diseases-10 (S92.301 (A, B), S92.302 (A, B), S92.309 (A, B), S92.351 (A, B), S92.352 (A, B), S92.353 (A, B), S92.354 (A, B), S92.355 (A, B), S92.356 (A, B)) was used to query the data from the institution’s data repository. Eligible participants were adults (≥18 years) who underwent a complete three-view foot radiographic series (anteroposterior, oblique, and lateral). Cases (Fx, or fracture) were patients with an isolated 5MT fracture confirmed on these views. We excluded cases with fractures involving additional foot regions; the presence of hardware, a cast, or other imaging artifacts; bone cysts; or incomplete radiograph views. Controls (NoF, or no fracture) were adults with no evidence of foot fracture on the same radiographic series. We excluded potential controls if they were younger than 18 years; had any foot fracture; showed severe deformity (such as hallux valgus, hammer toes, metatarsus adductus, or Charcot disease); had advanced degenerative changes or acute soft-tissue injury; had hardware, a cast, or imaging artifacts; had bone cysts; or lacked a complete three-view series.

Data collection and preparation

Three experienced orthopedic researchers reviewed the dataset for inclusion/exclusion criteria. Radiographs, along with CT scans, MRIs (where available), and clinical radiological notes, were carefully examined to confirm diagnoses. Once eligibility was confirmed, radiographs were extracted from the institution’s Electronic Health Records (EHR) system and saved in Portable Network Graphics (PNG) format. SP photography was used to capture radiograph images, employing different Android and iOS SPs and screens to ensure variability, which was also considered a manual augmentation technique. All photographs were taken in a dark room, with screens at maximum brightness and the cellphone positioned approximately 20 centimeters from the screen. 

**Figure 1:**
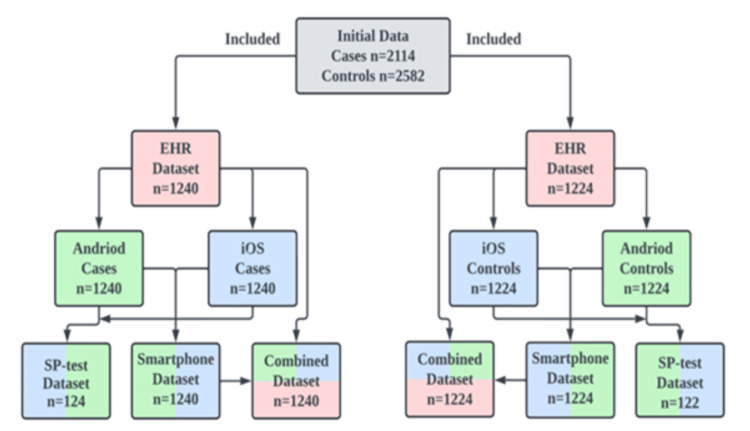
Study enrollment flowchart. Orange: Data retrieved directly from the Electronic Health Record (EHR); Green: Android device captured images of the EHR database; Blue: iOS device captured images of the EHR database.

For the Fx set, we created an EHR dataset consisting of three-view foot radiographs (anteroposterior, lateral, and oblique) from 1,240 patients. Two versions of the EHR dataset were created by photographing radiographs displayed on computer screens using SP cameras - one set with an Android device and the other with an iOS device (Figure 1). Five percent of each version was reserved for final testing, ensuring no duplicates for the SP-test. The SP-test images were also removed from the original EHR dataset. An SP dataset was then created by combining 50% of the remaining images from each version, without duplicate patients. Lastly, 50% of the SP and EHR datasets were mixed to build the combined dataset, avoiding duplicates. The same process was followed for the NoF group, including a dataset of 1,224 healthy individuals (Figure 1). 

In the preprocessing phase, the images were resized to a uniform dimension of 600 × 600 pixels, with the three color channels - red, green, and blue - aligning with common practices in medical image processing, where consistent image dimensions are essential for model input [20]. Normalization of pixel values to the range (0, 1) was performed to standardize the data, a step that is important in deep learning to ensure model convergence and performance optimization [21]. 

Model development 

Three convolutional neural networks (CNNs) were designed for the classification task using the Keras library and TensorFlow platform. The ResNet 152-v2 architecture was used as the base of the models, with an additional dense layer with 1,024 filters (with rectified linear unit (ReLU) activation), a dropout layer with a 0.4 dropout rate, and a final dense layer with sigmoid activation added for binary classification [22]. The models were compiled using the Adam optimizer with an initial learning rate of 1 × 10^-4^, and binary cross-entropy was used as the loss function [23]. After training for 50 epochs, the last 20 layers of the ResNet model were unfrozen, and the model was retrained with a learning rate of 1 × 10^-6^. Additional layers were trained if training accuracy did not improve [24]. The models were trained using a 90:10 training and validation split on their respective datasets and then tested on the SP-test dataset. This allowed for comprehensive training and performance evaluation. 

Statistical analysis 

Data analysis was conducted using IBM SPSS Statistics for Windows, Version 28 (Released 2021; IBM Corp., Armonk, NY, USA). We assessed the normality of continuous data using the Kolmogorov-Smirnov and Shapiro-Wilk tests. Depending on the distribution of the data, the Mann-Whitney U test was applied for comparisons. Results are presented as median (interquartile range (IQR)) and percentages (%). The performance of the models was evaluated based on key metrics such as the area under the receiver operating characteristic curve (AUROC), sensitivity, specificity, positive predictive value (PPV), negative predictive value (NPV), and Youden’s J index. A p-value of ≤0.05 was considered statistically significant. 

## Results

A total of 1,240 records with a median age of 56 (36, 68) years and 1,224 records with a median age of 62 (51, 72) years were included in the Fx and NoF groups, respectively (p < 0.001). Although most participants in both groups were White, there was a significant difference in the racial composition of the study groups (p < 0.001; Table 1)

**Table 1:** Demographic characteristics of the study groups. Data is presented as median (IQR) or percentage. ^a^ Mann-Whitney U test; ^b^ Chi-square test. IQR, Interquartile Range

Characteristics		Cases	Controls	p-value
Age (years)		56 (36, 68)	62 (51, 72)	<0.001^a^
Gender (female)		75.5%	72.1%	0.174^b^
Race	White group	84.8%	92.2%	<0.001^b^
	Non-white group	15.2%	7.8%	

**Figure 2:**
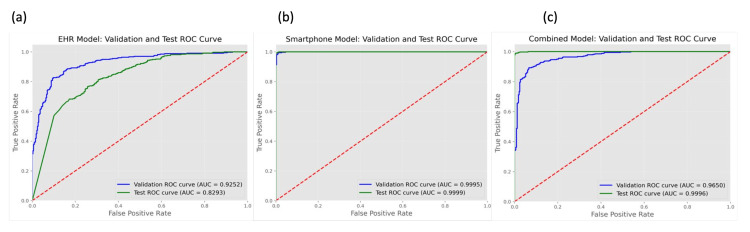
Comparison of receiver operating characteristic (ROC) curves for study models, evaluated on both validation and smartphone datasets (SP-test). (a) Electronic Health Record (EHR) model, developed on data directly retrieved from the EHR system; (b) Smartphone model, developed on smartphone-captured images of EHR data; (c) Combined model, developed using both EHR and smartphone datasets.

Our models were evaluated for the detection of 5MT fractures on X-rays in various views. All three models showed high performance when evaluated on their validation datasets, with the SP model achieving the best performance, with an AUROC of 0.99 and a Youden’s J of 0.95 (Table 2 and Figure 2). On the SP-test dataset, the SP and combined models maintained high performance, while the EHR model showed a decline, with an AUROC of 0.83 and a Youden’s J of 0.88 (Table 3 and Figure 2). 

**Table 2:** Performance metrics of the study models tested on the original validation datasets, with 95% confidence intervals shown in parentheses. EHR: Electronic Health Record; SP: Smartphone; AUROC: Area Under the Receiver Operating Characteristics Curve; PPV: Positive Predictive Value; NPV: Negative Predictive Value

Models	AUROC	Sensitivity	Specificity	Youden Index	F1 Score	Accuracy	PPV	NPV
EHR	0.92 (0.90-0.94)	0.82 (0.79-0.85)	0.90 (0.87-0.92)	0.72 (0.69-0.77)	0.85 (0.83-0.88)	0.86 (0.83-0.88)	0.89 (0.86-0.91)	0.83 (0.80-0.86)
SP	0.99 (0.99-1.00)	0.96 (0.95-0.98)	0.99 (0.99-1.00)	0.95 (0.93-0.97)	0.98 (0.97-0.99)	0.98 (0.97-0.99)	0.99 (0.99-1.00)	0.96 (0.95-0.98)
Combined	0.96 (0.95-0.97)	0.81 (0.78-0.84)	0.97 (0.96-0.98)	0.78 (0.73-0.83)	0.88 (0.86-0.90)	0.89 (0.86-0.91)	0.96 (0.95-0.98)	0.83 (0.81-0.86)

**Table 3:** Performance metrics of study models, evaluated on an independent smartphone-captured dataset (SP-test) not used during model development, with 95% confidence intervals shown in parentheses. EHR: Electronic Health Record; SP: Smartphone; AUROC: Area Under the Receiver Operating Characteristics Curve; PPV: Positive Predictive Value; NPV: Negative Predictive Value

Models	AUROC	Sensitivity	Specificity	Youden Index	F1 Score	Accuracy	PPV	NPV
EHR	0.83 (0.80-0.85)	0.98 (0.97-0.99)	0.90 (0.87-0.92)	0.88 (0.85-0.91)	0.85 (0.83-0.88)	0.86 (0.83-0.88)	0.89 (0.86-0.91)	0.83 (0.80-0.86)
SP	0.99 (0.99-1.00)	0.98 (0.98-0.99)	0.99 (0.99-1.00)	0.98 (0.97-0.99)	0.99 (0.99- 1.00)	0.99 (0.99-1.00)	0.99 (0.99-1.00)	0.98 (0.98-0.99)
Combined	0.99 (0.99-1.00)	0.97 (0.96-0.98)	0.99 (0.99-1.00)	0.96 (0.94-0.98)	0.98 (0.98-0.99)	0.98 (0.98-0.99)	0.99 (0.99-1.00)	0.97 (0.96-0.98)

## Discussion

Plain radiographs are the first-line diagnostic tool for detecting various orthopedic injuries. Despite their widespread use, interpretation challenges often arise, particularly in RL settings or when handled by inexperienced clinicians or care providers. This study assessed the diagnostic accuracy of AI algorithms for detecting 5MT fractures on SP-captured images of radiographs, developed using combinations of datasets. Our findings showed that AI models trained exclusively on SP images achieved superior diagnostic performance, highlighting the potential of tailored training for SP-based diagnostic tools.

The application of AI in orthopedics, especially for fracture detection, is a well-studied area that has shown performance comparable to human raters. Vertebral, hip, and upper extremity fractures are among the most extensively researched areas [25]. The majority of these studies utilized plain radiographs as the modality of interest, resulting in a pooled sensitivity of 94% and a specificity of 92% for fracture detection [26]. However, the use of deep learning for fracture detection in the foot area remains comparatively underexplored [27]. The complex anatomy of the foot and the subtle presentation of certain fractures pose unique challenges. Nevertheless, emerging research has begun to address this gap. Kim et al. evaluated the efficiency of different CNNs for foot fracture detection and reported an AUROC of up to 0.95 using ensemble methods [28]. Their study, however, included a relatively small sample of 436 single-view images combining different fractures in the foot area. Similarly, Wang et al. conducted a study using 1,151 lower extremity radiographs to investigate the application of deep learning in detecting stress fractures and grading their severity [29]. They reported an overall AUROC of 0.94, missing only 9.7% of lower-grade fractures, compared to 39.9% missed by human raters. However, none of these studies provided a subgroup analysis of each specific fracture in the foot area, likely due to a lack of sufficient sample size. In our study, we addressed this limitation by specifically training models to detect 5MT fractures using a larger sample size. Our models achieved an AUROC of up to 0.99, sensitivity of 0.96, and specificity of 0.99, confirming the results of previous studies and underscoring the potential of deep learning algorithms in accurately detecting specific foot fractures.

With over three billion users worldwide, SPs are among the most accessible technologies today [30]. Given the increasing importance of diagnosing, incorporating it into SPs is an effective way to enhance their utility, accessibility, and improve healthcare quality. For instance, Yang et al. developed an algorithm using 1,819 SP-acquired images of thick blood smears to detect malaria, achieving an AUROC of 98.39% [31]. More relevant to our study, Rangarajan and Ramachandran developed an SP application to diagnose COVID-19 using images of chest radiographs [32]. Their best-performing model reported a positive likelihood ratio of 27.3 when evaluated on SP-based images of radiographs comprising 271 healthy, 441 pneumonia, and 48 COVID-19 cases. However, due to the small size of their dataset, factors such as variations in camera quality, screen quality, and user technique pose significant risks to reliable performance outside controlled environments. Furthermore, they employed publicly available datasets and techniques like data augmentation and generative adversarial networks (GANs) to synthesize new data and address the issue of sample size limitation for their training dataset. While these techniques are invaluable, they may lead to overfitting or generate unrealistic image features that do not generalize well to real-world scenarios, especially in clinical diagnosis [33]. In our study, we aimed to address these issues by including a larger sample size and creating SP datasets using various brands of cellphones, screens, and users to replicate real-world scenarios. Our results demonstrated higher performance of the SP and combined models when tested using SP images. However, the SP and combined models showed higher performance even when tested on their own validation sets. One possible reason could be that the changes made to the images by SPs’ computational photography, including tone-mapping, local contrast enhancement, and sharpening, could potentially increase the edge contrast around the fracture lines. After down-sampling to 600 × 600, these enhanced edges may make the signal of interest more linearly separable for the models. Nevertheless, these results show the added value of utilizing SP images for model training when developing smart device applications.

This study utilizes data from three hospitals within a single geographic region, which may limit generalizability to settings with different patient demographics, clinical workflows, and imaging hardware or software. As a result, external validation across independent health systems and vendors will be necessary to confirm real-world applicability. To reduce confounding from extreme anatomy and imaging artifacts, we excluded severe deformities; while this improves internal validity, it narrows the clinical spectrum represented and may limit performance in patients with pronounced deformity, post-traumatic sequelae, or advanced Charcot changes. In addition, the SP dataset was created by re-photographing clinical displays, a workflow that mirrors plausible point-of-care use but may introduce device- and pipeline-specific effects that differ from other environments. Regardless of these constraints, our cohort is larger than many prior reports, and we intentionally introduced diversity when constructing the SP set (multiple devices, conditions, and manual augmentation) to better approximate real-world variability. These steps may partially mitigate, though not eliminate, concerns about generalizability.

## Conclusions

Deep learning models demonstrate superior performance in detecting 5MT fractures from SP-captured images when trained on specific datasets. Our results suggest that developing models tailored for smart device-based imaging could offer a practical diagnostic solution in medical settings with limited access to specialized expertise or advanced imaging equipment. Nonetheless, further prospective validation is necessary to confirm these models’ utility and robustness in clinical applications.
